# Biomarker Development for Identifying Mud Loach (*Misgurnus mizolepis*) Origin Country Using Untargeted Metabolite Profiling

**DOI:** 10.3390/life13112149

**Published:** 2023-10-31

**Authors:** Hyunsuk Kim, Jiyoung Shin, Junho Yang, Yikang Sim, Ji-Young Yang

**Affiliations:** Department of Food Science & Technology, Pukyong National University, Busan 48513, Republic of Korea

**Keywords:** *Misgurnus mizolepis*, geographical origin, LC-QTOF/MS, metabolite, multivariate analysis

## Abstract

Mud loach (*Misgurnus mizolepis*) has long been consumed in Korea. Recently, Chinese mud loaches were replaced with expensive Korean mud loaches, owing to taste and preference. Such issues occur in aquatic food distribution processes, leading to inferior food delivery. Previously, a study was conducted to confirm the origin of mud loaches using genetic analysis. However, untargeted metabolites profiling of mud loaches has not been reported. Untargeted metabolomics provides information on the overall metabolic profiling of a sample, allowing the identification of new metabolites. Here, we analyzed the metabolites of mud loaches of different geographical origins using liquid chromatography (LC)-quadrupole-time-of-flight mass spectrometry (MS). Orthogonal partial least squares discriminant analysis from LC/MS datasets showed a clear distinction between Korean and Chinese mud loaches, and univariate statistical analysis showed significantly different metabolites between them. N-acetylhistidine and anserine were selected as biomarkers for geographical origin discrimination using the receiver operating characteristic curve. N-acetylhistidine and anserine levels were significantly higher in Chinese than in Korean mud loaches. These results indicate that metabolic analysis can be used to discriminate between the geographical origins of mud loaches, curtailing the inadvertent substitution of mud loaches from different regions.

## 1. Introduction

Mud loach (*Misgurnus mizolepis*), a species belonging to the family Cobitidae, has long been consumed in Korea owing to its nutritional excellence and good flavor. Its habitat is sand or muddy bottoms in the middle and lower reaches of rivers, and it is distributed not only in Korea but also in China and Taiwan [1,2]. However, the domestic aquaculture industry has stalled because of a lack of natural seeding and environmental degradation. Therefore, the market for Chinese fish species is showing an increasing trend in Korea [3]. Recently, there was a case where Chinese mud loaches were replaced with expensive Korean mud loaches, because of the fact that the latter is preferred for its taste. It is difficult to distinguish between the two owing to their high degree of morphological similarity. Additionally, low-quality mud loaches have excessive amounts of ofloxacin and enrofloxacin, used as animal antibiotics. Therefore, it is crucial to distinguish between the geographical origins of Korean and Chinese mud loaches.

Metabolomics is the comprehensive and quantitative analysis of metabolite interactions in biological systems [4]. Food metabolomics has been used to identify the geographical origins of products, analyze food ingredients, and determine authenticity by identifying and monitoring low-molecular-weight metabolites [5,6,7,8]. Metabolic analysis techniques are based on mass spectrometry (MS), including liquid chromatography (LC)/MS, gas chromatography, and nuclear magnetic resonance (NMR) spectroscopy [9,10,11,12]. In data processing, multivariate data analysis, including principal component analysis (PCA) and orthogonal partial least squares discriminant analysis (OPLS-DA), can be used to extract information by simultaneously analyzing multiple variables. PCA is used for extracting distributed variables by reducing their dimensions [13] and expressing them as orthogonal variables, thereby simplifying the dataset size [14]. OPLS-DA is used to identify biomarker candidates by excluding independent variables irrelevant to the data while simultaneously considering dependent variables to find relationships between groups [15,16]. Thus, supervised OPLS-DA is a prediction and regression analysis method that can distinguish between two groups [17]. The evaluation of a model’s classification performance across multiple thresholds can be measured using receiver operating characteristic (ROC) curve and area under the ROC curve (AUC) values. The analysis of ROC curves is widely accepted as the standard method for performance assessment. These curves display the changing sensitivity and specificity levels for varying classification decision boundaries, which are dependent on the range of biomarker scores available [18].

In international trade, food origin regulations are becoming stricter, and management strategies are being embodied. Therefore, the identification of the geographical origin of food is becoming more important as the awareness of consumers regarding the country of origin increases. Since it is possible to identify metabolites according to geographic factors, many studies on determining food origins are in progress [19]. Recently, metabolite profiling of perilla, sesame, goji berries, and tobacco leaves has been reported [20,21,22]. Owing to the specificity of climate and nutrition, studies on agricultural products are progressing in various ways. In the case of livestock products, the origin of beef has been determined using 1H NMR-based metabolomics [23]; however, studies on the identification of the origin of seafood using metabolomes are insufficient. Although a method using a primer targeting the cytochrome c oxidase subunit I gene region of mitochondrial DNA has been reported to discriminate the origin of mud loaches [24], an identification biomarker using metabolomics has not yet been developed.

This study aimed to use a metabolomic approach to distinguish between Korean and Chinese mud loaches. An untargeted analysis using LC-QTOF/MS and multivariate statistical analyses were used for metabolic profiling to determine the origin of the fish. The discriminant model was used to develop a biomarker that could determine the geographical origin of the mud loaches.

## 2. Materials and Methods

### 2.1. Sample Collection and Preparation

**Figure 1:**
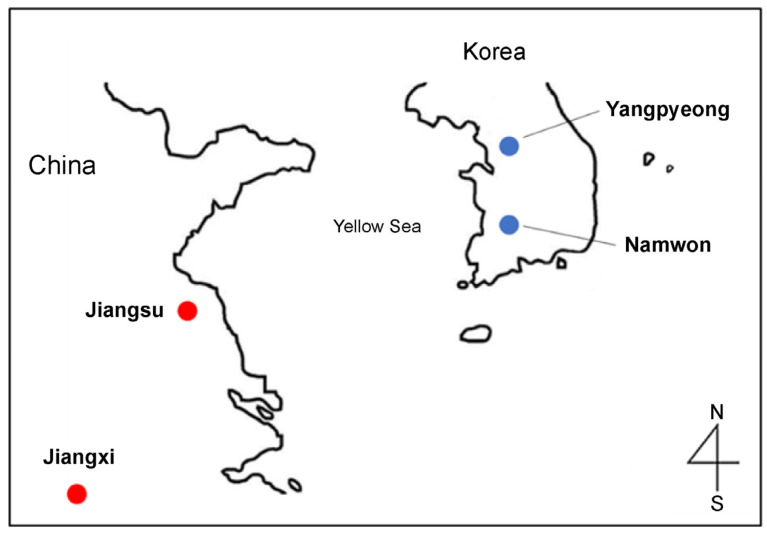
Geographical origins of the mud loach in Korea and China.

**Figure 2:**
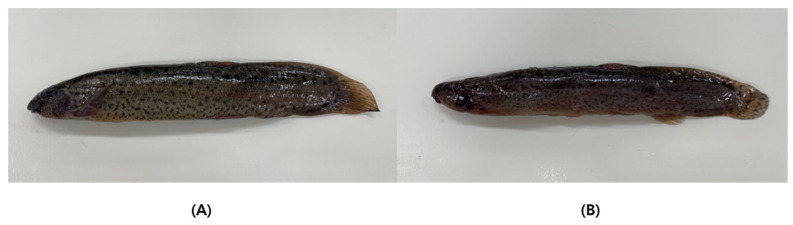
Korean (**A**) and Chinese (**B**) mud loaches.

In 2022, twelve Korean and twelve Chinese mud loaches were obtained from a local fish market in Busan and an aquaculture farm. Both mud loach samples from Korea and China were cultivated inland and included the areas of Yangpyeong (Gyeonggi-do), Namwon (Jeollanam-do), Jiangsu, and Jiangxi (Figure 1). Images of the mud loaches are presented in Figure 2 and sample information is in Table 1. Tissue of fish was taken after anesthesia. The animal study protocol was reviewed and approved by the Pukyung National University-Institutional Animal Care and Use Committee on ethical procedures and scientific care (approval number: PKNUIACUC-2022-30). Muscle tissue was collected and lyophilized for 48 h in a freeze dryer (cooling trap HC3110, Bio-Medical Science Co., Ltd., Seoul, Republic of Korea). All samples were vacuum packed and stored at −80 °C until further analysis.

### 2.2. Metabolite Extraction

The mud loach samples (20 mg) were homogenized using a Mixer Mill MM 400 (Retsch GmbH, Haan, Germany) at 28 Hz for 1 min and then mixed with 1.6 mL of methanol:isopropyl alcohol:water (3:3:2, *v*/*v*/*v*) as the extraction solvent. After vortexing, the mixtures were sonicated for 10 min and centrifuged for 15 min at 16,800× *g* and 4 °C. The supernatant was pipetted into a fresh tube and dried using a speed vacuum concentrator (HyperVac, Gyrozen Co., Ltd., Gimpo, Republic of Korea). The dried samples were reconstituted in 100 μL water and the extracts filtered using a 0.2 μm polytetrafluoroethylene (PTFE) filter (Advantec Toyo Kaisha, Tokyo, Japan).

### 2.3. LC-Quadrupole-Time-of-Flight (QTOF)/MS Analyses

Metabolite profiling was performed using an Acquity UPLC I-Class PLUS System (Waters, Milford, MA, USA) equipped with QTOF/MS (Synapt XS, Waters, Milford, MA, USA). Chromatographic separation was performed using the ACQUITY UPLC BEH C8 column (Waters; 2.1 × 100 mm, 1.7 μm) at a flow rate of 0.3 mL/min. The mobile phase consisted of 0.1% formic acid in water (phase A) and 0.1% formic acid in acetonitrile (phase B). The gradient elution program was applied as follows: 0–0.1 min, 0.5% B; 0.1–10 min, 80% B; 10–10.1 min, 99.5% B; 10.1–12 min, 99.5% B; 12–12.1 min, 0.5% B; and 12.1–15 min, 0.5% B. The injection volume was 1 μL. A QTOF/MS analysis was performed using an electrospray ionization (ESI) source in the positive and negative ion modes with a mass range from 50 to 1200 *m*/*z*. The MS parameters were as follows: source capillary, 3 kV; sampling cone, 60 V; source temperature, 120 °C; desolvation temperature, 350 °C; cone gas, 20 L/h; desolvation gas, 650 L/h; and MS-elevated collision energy, 20–45 V. To estimate the metabolite analysis stability, quality control (QC) samples containing the same amount of all samples were inserted. QC samples are also utilized to differentiate between outlier samples that may be biologically distinct from the norm, or merely part of the general systemic variability. Their inclusion enables the system to effectively evaluate the quality of the data produced [25].

### 2.4. Data Processing

Peak alignment and picking of the MS data were performed using Progenesis QI (v.2.4, Waters) software. The metabolite matrix was obtained according to the retention time (RT) and mass-to-charge ratio (*m*/*z*). To distinguish between the geographic origins of the two mud loach groups, MetaboAnalyst v5.0 (www.metaboanalyst.ca, accessed on 6 December 2022) was used for the multivariate statistical analysis. A PCA and OPLS-DA were performed for clustering between groups and to identify potential biomarkers among the detected metabolites, respectively. Qualification of the discriminant model was evaluated using R2Y (goodness of fit measure) and Q2 (predictive ability) values. The data for each ion were normalized using Pareto scaling. Heatmap visualization was performed based on Pearson’s correlation. The discriminating metabolites were selected based on a Variable Importance in Projection (VIP) score > 1 in the OPLS-DA model and a *p*-value < 0.05 in the *t*-test. An ROC analysis was used to identify biomarkers. Subsequently, metabolite structure identification was performed in databases—Kyoto Encyclopedia of Genes and Genomes (KEGG), Chemical Entities of Biological Interest (ChEBI), the Human Metabolome Database (HMDB)—by matching 5 ppm of precursor tolerance and 5 ppm of fragment tolerance.

### 2.5. Chemicals and Reagents

N-acetylhistidine was purchased from Sigma-Aldrich (St. Louis, MO, USA), anserine from MedChemExpress (Monmouth Junction, NJ, USA), and high-performance LC (HPLC)-grade methanol, isopropyl alcohol, acetonitrile, formic acid, and water from Honeywell Burdick & Jackson (Muskegon, MI, USA).

## 3. Results

### 3.1. Metabolite Profiling of the Mud Loach

Metabolite profiles were acquired from the mud loaches that were cultivated in the two regions, Korea and China. To discriminate the geographic origin of the mud loaches, metabolite profiling was performed using LC-QTOF/MS. Putative metabolite identification was based on RT and *m*/*z*. A total of 21,772 metabolite ion features—15,013 and 6759 in the positive and negative ion modes, respectively—were extracted from Korean and Chinese samples. QC samples were analyzed using a PCA and multivariate data analysis. QC mud loach samples were clustered, indicating that the dataset was valid and stable.

### 3.2. PCA and OPLS-DA for Geographic Differentiation of the Mud Loach

**Figure 3:**
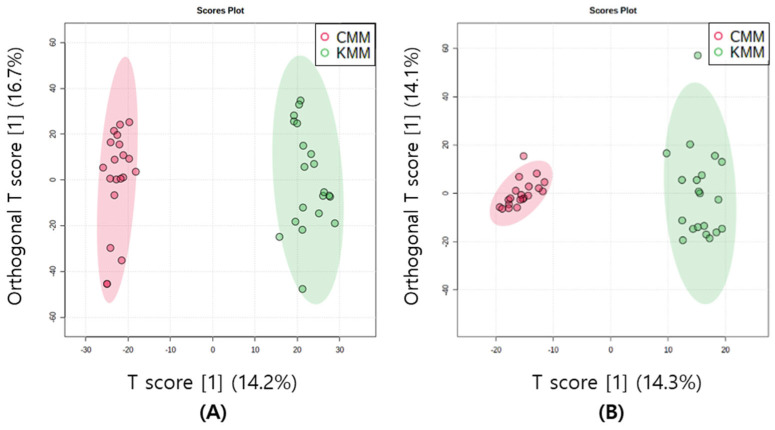
Orthogonal partial least squares discriminant analysis (OPLS-DA) scores plots of mud loach samples in positive (**A**) and negative (**B**) ion mode derived from liquid chromatography/mass spectrometry (LC/MS).

The multivariate statistical method is a technique that facilitates the clustering of multiple metabolomic data based on common features. This allows for the identification of distinctions between samples and the determination of correlations between variables. Unsupervised PCA score plots were constructed for the two groups according to their geographical origin in both ion modes. The first two components of the PCA model explained 44.6 and 49.4% of the variance in the positive and negative ion modes, respectively. In both modes, clustering appeared, although there was an origin overlap between the two samples. Supervised OPLS-DA score plots were used to confirm a clearer separation. In metabolomics, cross-validation through R2 and Q2 parameters is frequently performed. R2 measures the goodness of fit, and the closer the value is to 1, the better the explanation of the data by the model. Q2 measures the predictive power of the model, and a value closer to 1 indicates perfect predictability [26]. According to the SIMCA guidelines, when Q2 is greater than 0.5, moderate predictability is confirmed [27]. To enhance geographic discrimination in mud loaches, OPLS-DA was used to determine metabolomic differences. The OPLS-DA model with 0.90 for R2Y and 0.86 for Q2 in positive ion mode, and 0.92 for R2Y and 0.9 for Q2 in negative ion mode was suitable and well validated. The Korean and Chinese mud loaches were distinctly divided and clustered into two groups by T1, both positive and negative ion modes (Figure 3).

### 3.3. Identification of Differential Metabolites as Biomarkers

**Figure 4:**
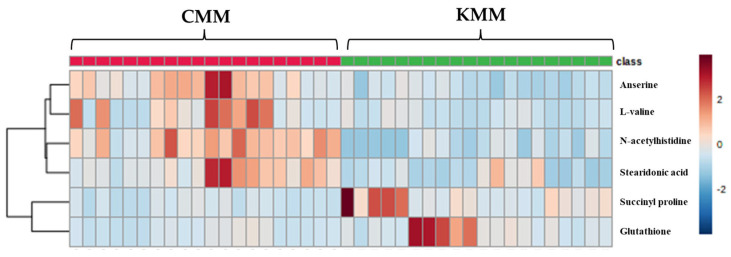
Heatmap of different metabolites between Korean (KMM) and Chinese (CMM) mud loaches.

The VIP score was used to indicate the variable contribution, divided into two groups. Potential biomarker metabolites were selected based on VIP values >1 from the OPLS-DA [28]. Additionally, the *p*-value < 0.05 and foldchange were evaluated. A total of six ion features were confirmed, and metabolite identification was performed—which had high discrimination overall for amino acid and fatty acid metabolites—using the database (Table 2). All six compounds were detected in positive ion mode. The identified metabolites were anserine ([M + H]^+^ *m*/*z* 241.1304), succinyl proline ([M + H]^+^ *m*/*z* 216.0871), glutathione ([M + H]^+^ *m*/*z* 611.1448), L-valine ([M + H]^+^ *m*/*z* 118.0865), N-acetylhistidine ([M + H]^+^ *m*/*z* 198.0088), stearidonic acid ([M + H]^+^ *m*/*z* 277.2164). N-Acetylhistidine was top-ranked in the VIP score (2.07) and fold change (4.06). A cluster analysis is a user-friendly visualization tool that utilizes colors to represent data values; therefore, it is possible to configure indicators corresponding to rows and columns and to identify characteristics with high or low indicator values. Blue-filled cells have a low compound content in the sample, while red-filled cells have a high content. Additionally, the darker the color, the higher the correlation. In the heat map, the metabolites of Korean and Chinese mud loach samples were separated into two distinct groups, forming clusters (Figure 4). Glutathione, and succinyl proline were significantly higher in the Korean samples; however, N-acetylhistidine, anserine, stearidonic acid, and L-valine were significantly higher in the Chinese samples.

**Figure 5:**
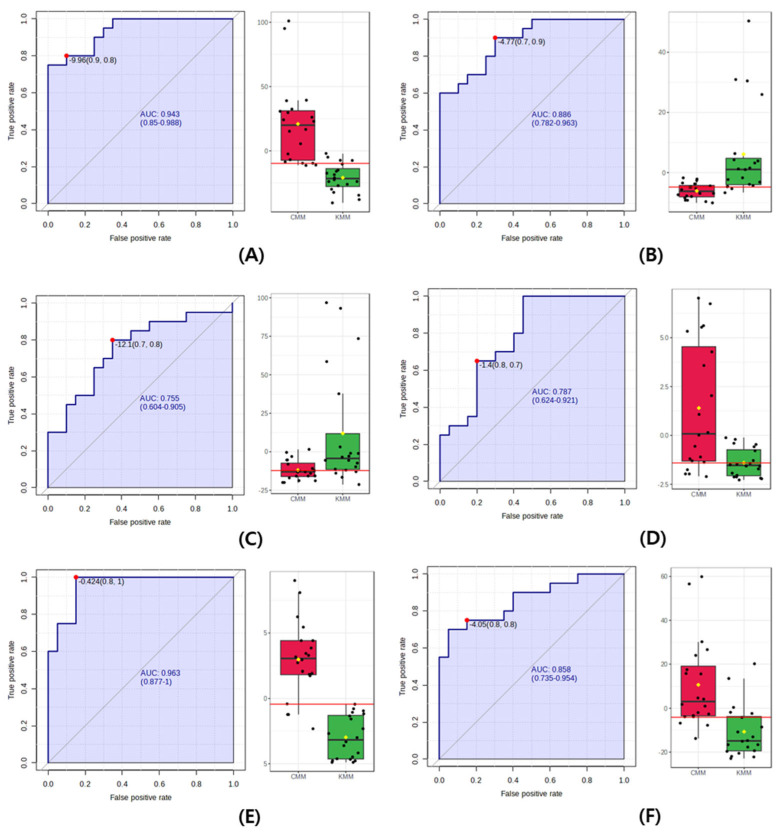
Receiver operating characteristic (ROC) curve to discriminate between Korean (KMM) and Chinese mud loach (CMM). (**A**) anserine; (**B**) succinyl proline; (**C**) glutathione; (**D**) L-valine; (**E**) N-acetylhistidine; (**F**) stearidonic acid. In the figure, the black dots are the individual contents of the samples, and the red and green boxes are the significant average values of CMM and KMM.

Finally, the ROC curve, which expresses the performance of a discriminant model, was used to identify the origin biomarkers in the mud loaches. The closer the AUC value—calculated as the area under the ROC curve—is to 1, the better the performance of the model [29]. A rough guideline for evaluating the usefulness of a biomarker based on AUC values is used as follows: 0.9–1.0 = excellent; 0.8–0.9 = good; 0.7–0.8 = fair; 0.6–0.7 = poor; and 0.5–0.6 = fail. As shown in Figure 5, the AUC value of succinyl proline, glutathione, L-valine, and stearidonic acid was 0.87, 0.76, 0.79, and 0.86. In the guideline, AUC values did not achieve the “excellent” criterion for use as a biomarker, with values below 0.9. However, the AUC value of N-acetylhistidine was 0.96, indicating the excellent precision of discriminating Korean and Chinese mud loaches. Additionally, the AUC of anserine provides a good quality score, representing the overall performance. The AUC value of the multivariate ROC curve for the two selected metabolites was 0.99. Therefore, N-acetylhistidine and anserine were proposed as potential biomarkers to determine the geographical origin of the mud loaches.

N-acetylhistidine has a parent ion of [M + H]^+^ 198.0088 *m*/*z* and was detected at 6.02 RT. N-acetylhistidine, an imidazole compound, is L-histidine with an acetyl substituent on the alpha nitrogen and can be biosynthesized from L-histidine and acetyl-Coa by the enzyme histidine N-acetyltransferase. N-acetylhistidine is a major non-protein nitrogen component in vertebrate skeletal muscles [23], and decreases in muscle tissue under prolonged starvation [30], but is fully recovered after the resumption of feeding. Additionally, increasing water temperatures increases the N-acetylhistidine content of fish [31]. This supports the hypothesis that the N-acetylhistidine level is higher in Chinese samples because the water temperature is higher in China than in Korea.

Anserine has a parent ion of [M + H]^+^ 241.1304 *m*/*z* and was detected at 0.82 RT. Anserine, a dipeptide composed of β-alanine and 3-methyl-L-histidine, is present in the skeletal muscle of fish [32,33]. Like N-acetylhistidine, it is an imidazole-related compound that acts as an H+ buffer, neurotransmitter, non-enzymatic free radical scavenger, antioxidant, and blood sugar regulator and is involved in the histidine metabolism pathway. The varying anserine content in the genus *Oncorhynchus* is affected by muscle buffering capacity and swimming activity but not by variations in the plant protein content in fish diets; that is, anserine deficiency is related to muscle pH [34]. Chinese mud loaches are transported to Korea tied in a plastic bag filled with water. Therefore, the physical environment during the import and distribution process may contribute to the difference in the anserine levels of Korean and Chinese mud loaches.

## 4. Discussion

Mud loaches imported into Korea are all from China. Chinese mud loaches account for a large portion of the Korean market; therefore, there is a possibility that Korean fish prices may be affected by an increase in Chinese imports. Jiangxi and Jiangsu provinces were selected for metabolic profiling of mud loaches imported from China. Jiangxi has the highest proportion of loach production by region. Jiangsu was next, and is being used as a gathering place for distribution before export to Korea.

Previously, there was a study to confirm the origin of mud loaches using genetic analysis [24]. However, untargeted metabolites profiling of mud loaches has not been reported. Untargeted metabolomics provides information on the overall metabolic profiling of a sample. This allows the identification of new metabolites. Therefore, we analyzed the primary metabolites of mud loaches using LC-QTOF/MS.

Fish is a high-protein food and contains a variety of high-quality amino acids. Some amino acids and their metabolites are important regulators of key metabolic pathways required by a variety of organisms. In various fish species, essential and non-essential amino acids are involved in the maintenance, growth, reproduction and immunity of aquatic animals [35]. In our study, metabolites related to amino acids and fatty acids were detected in mud loaches. The identified metabolites were anserine, succinyl proline, glutathione, L-valine, N-acetylhistidine, and stearidonic acid. Among them, N-acetylhistidine showed significant differences between Korean and Chinese mud loaches. N-acetylhistidine is contained in fish muscles in large quantities, and N-acetylated derivatives of histamine-related compounds have been reported to occur in various tissues of vertebrates [36]. Changes in N-acetylhistidine levels are related to nutrient intake and changes in water temperature [31]. This is the assumed cause of the difference between Korean and Chinese mud loaches in our study. In addition, our study also showed differences in anserine. Anserine is a dipeptide found as an abundant non-protein nitrogen-containing compound [37]. It is reported to have a significant effect on muscle functions, such as pH buffering ability, antioxidant capacity, and increased Ca^2+^ sensitivity; these previous studies are consistent with our results [34].

In this study, pareto scaling was selected to extract optimal information. A study of the correlation between information found in different metabolites from different analytical platforms is important to validate the extracted information. Therefore, valuable information regarding the removal of extraneous metabolites should be obtained. Data preprocessing and scaling are essential to normalize metabolites and eliminate spurious sample-to-sample variability [38]. We determined the geographical origins of mud loaches cultivated in Korea and China using OPLS-DA. Supervised OPLS-DA score plots showed a clear distinction. VIP score plot was produced to identify biomarkers to discriminate the geographic origin of mud loaches. *p*-value was used to confirm the statistical significance level, and fold change was used as a measure to explain how much the amount varies between samples. Finally, Anserine, succinyl proline, glutathione, L-valine, N-acetylhistidine, and stearidonic acid were identified as putative metabolites for discrimination. These biomarkers were further validated through ROC curve analysis. AUC values of potential biomarkers ranged from 0.75 to 0.96. According to the guidelines for biomarker availability, N-acetylhistidine and anserine with an AUC value of 0.9 or higher were selected as the final biomarkers to be able to clearly distinguish the geographical origin of country. Therefore, our study suggests that N-acetylhistidine and anserine can be used as potential biomarkers for geographical discrimination of mud loaches.

Histamine is a biogenic amine that stimulates several histamine receptor types. The major pathway of histidine metabolism in fish muscle is enzymatic decarboxylation of histamine [39]. Histidine and anserine are imidazole-related compounds and are the major non-protein nitrogen components occurring in the skeletal muscle of vertebrates. N-Acetylhistidine is a histidine derivative that is L-histidine having an acetyl substituent on the alpha-nitrogen. N-Acetylhistidine is synthesized from L-His and acetyl-Coa by the histidine acetyltransferase, and hydrolyzed to histidine by the anserinase. In the present study, the content of N-acetylhistidine in Chinese mud loaches was significantly higher than that of Korean mud loaches. Live fish do not feed during transportation. In addition, a previous study showed that the content of N-acetylhistidine in the muscles of mud loaches decreases as the starvation state is maintained. Another reason for the difference in compound content is that metabolites are affected by the geographical environment. As the water temperature increases, the content of N-acetylhistidine increases [31], and the water temperature in inland China is higher than that of the corresponding farms in Korea. Anserine is involved in histidine metabolism and is a derivative of methylated carnosine. Anserine is closely related to pH with muscle buffering capacity. In addition to gill respiration, mud loaches also perform intestinal respiration. However, during transportation, many individuals at a time proceed in a tight space with insufficient air. Therefore, as shown in our results, it is suspected that there will be a difference in anserine content between Chinese and Korean mud loaches.

Our study identified two distinct markers, N-acetylhistidine and anserine, that could discriminate the geographical origins of Korean and Chinese mud loaches. The variability of geographical sources was confirmed by selecting representative farms from Korea and the area that imports the most from China. A limitation of our study is that metabolomic variability was not evaluated according to climatic changes, since samples from the same season were analyzed. It is suggested that analyzing samples from different seasons may have different results. Most organisms have mechanisms that change according to environmental factors. Particularly, changes in temperature, geography, season, and water stress can both increase and decrease secondary metabolites by up to 50% [40]. In addition, metabolism, such as the synthesis of carbohydrates, proteins, and lipids change according to biological rhythms in fish [41]. Mackerel tuna (*Euthynnus affinis*) showed a difference in the expression level of lipid metabolism in response to exogenous rhythm changes, such as different weather on sunny and cloudy days [42]. Climatic parameters are expected to affect origin determination performance. Therefore, our research should consider climatic conditions, a necessary factor to distinguish geographical origins. Further research will be needed to confirm the variability according to climate by sampling each of the four seasons. Our study included analysis on many compounds at once using non-targeted metabolomic profiling. Therefore, further qualitative and quantitative analysis of potential biomarkers present in samples using targeted metabolome methods will be required. This will provide reliable information to determine the country of origin of mud loaches from Korea and China.

## 5. Conclusions

In this study, we identified mud loach metabolites from different geographical origins—Korea and China—using LC-QTOF/MS. A multivariate statistical analysis was used to select the metabolites with significant differences between the two groups. Additionally, these metabolites were confirmed and verified as biomarkers through quantitative analysis. A model for discriminating between different geographical origins was established using PCA and OPLS-DA. Considering the VIP score, fold-change, and *p*-value, N-acetylhistidine and anserine were selected as biomarkers to distinguish between the geographical origin of the two groups using the ROC curve. This study serves to highlight the use of metabolomic profiling studies in verifying the geographic origin of mud loaches. The results of this study are anticipated to mitigate potential confusion within distribution systems and curtail the inadvertent substitution of mud loaches from different regions. Additionally, this study will contribute to the development of aquatic metabolomics.

## Figures and Tables

**Table 1:** Korean and Chinese sample information.

	KMM		CMM	
	Total Length	Weight	Total Length	Weight
Spring	18.34 ± 0.34	32.41 ± 3.97	17.89 ± 0.39	36.70 ± 1.93
Summer	17.44 ± 0.23	26.61 ± 1.26	16.64 ± 0.89	26.14 ± 2.75
Autumn	14.14 ± 0.68	15.43 ± 2.35	15.14 ± 1.00	17.79 ± 0.94
Winter	13.14 ± 2.10	13.19 ± 3.49	14.04 ± 0.71	19.22 ± 4.88
Mean ± SD	15.77 ± 2.45	21.52 ± 8.25	15.95 ± 1.68	24.96 ± 8.06

**Table 2:** Different metabolites in Korean and Chinese mud loach.

No.	RT (min)	Identified Metabolites	Adduct	*m*/*z*	VIP Score	Fold-Change	*p*-Value
1	0.82	Anserine	[M + H]^+^	241.1304	1.70	2.18	2.43 × 10^−6^
2	0.87	Succinyl proline	[M + H]^+^	216.0871	1.30	3.31	1.35 × 10^−3^
3	1.83	Glutathione	[M + H]^+^	611.1448	1.30	3.31	1.35 × 10^−3^
4	1.96	L-Valine	[M + H]^+^	118.0865	1.33	4.00	6.19 × 10^−4^
5	6.02	N-Acetylhistidine	[M + H]^+^	198.0088	2.07	4.06	1.56 × 10^−9^
6	11.39	Stearidonic acid	[M + H]^+^	277.2164	1.45	2.56	2.40 × 10^−4^

RT, retention time; VIP, Variable Importance in Projection.

## Data Availability

The data presented in this study are available in this article.
